# Explainable Transformer-Based Deep Learning Model for the Detection of Malaria Parasites from Blood Cell Images

**DOI:** 10.3390/s22124358

**Published:** 2022-06-08

**Authors:** Md. Robiul Islam, Md. Nahiduzzaman, Md. Omaer Faruq Goni, Abu Sayeed, Md. Shamim Anower, Mominul Ahsan, Julfikar Haider

**Affiliations:** 1 Department of Electrical & Computer Engineering, Rajshahi University of Engineering & Technology, Rajshahi 6204, Bangladesh; mdnahiduzzaman320@gmail.com (M.N.); omaerfaruq0@gmail.com (M.O.F.G.); 2 Department of Computer Science & Engineering, Rajshahi University of Engineering & Technology, Rajshahi 6204, Bangladesh; abusayeed.cse@gmail.com; 3 Department of Electrical & Electronic Engineering, Rajshahi University of Engineering & Technology, Rajshahi 6204, Bangladesh; md.shamimanower@yahoo.com; 4 Department of Computer Science, University of York, Deramore Lane, York YO10 5GH, UK; 5 Department of Engineering, Manchester Metropolitan University, John Dalton Building, Chester Street, Manchester M1 5GD, UK; j.haider@mmu.ac.uk

**Keywords:** malaria parasite, image analysis, deep learning, transformer-based model, grad-cam visualization

## Abstract

Malaria is a life-threatening disease caused by female anopheles mosquito bites. Various plasmodium parasites spread in the victim’s blood cells and keep their life in a critical situation. If not treated at the early stage, malaria can cause even death. Microscopy is a familiar process for diagnosing malaria, collecting the victim’s blood samples, and counting the parasite and red blood cells. However, the microscopy process is time-consuming and can produce an erroneous result in some cases. With the recent success of machine learning and deep learning in medical diagnosis, it is quite possible to minimize diagnosis costs and improve overall detection accuracy compared with the traditional microscopy method. This paper proposes a multiheaded attention-based transformer model to diagnose the malaria parasite from blood cell images. To demonstrate the effectiveness of the proposed model, the gradient-weighted class activation map (Grad-CAM) technique was implemented to identify which parts of an image the proposed model paid much more attention to compared with the remaining parts by generating a heatmap image. The proposed model achieved a testing accuracy, precision, recall, f1-score, and AUC score of 96.41%, 96.99%, 95.88%, 96.44%, and 99.11%, respectively, for the original malaria parasite dataset and 99.25%, 99.08%, 99.42%, 99.25%, and 99.99%, respectively, for the modified dataset. Various hyperparameters were also finetuned to obtain optimum results, which were also compared with state-of-the-art (SOTA) methods for malaria parasite detection, and the proposed method outperformed the existing methods.

## 1. Introduction

**Figure 1:**
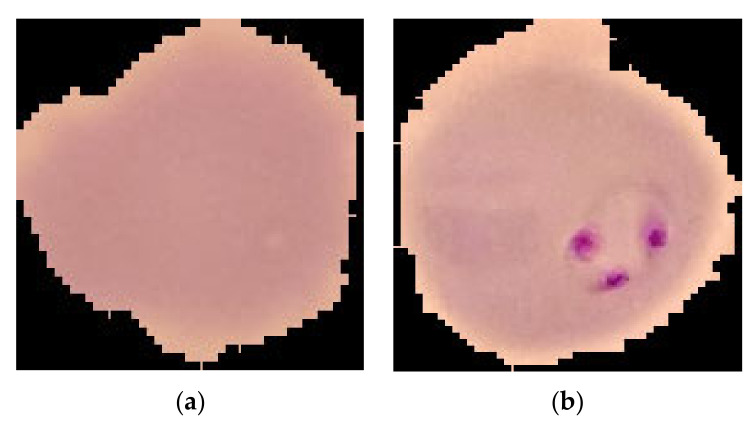
(**a**) Normal and (**b**) malaria-infected RBC images.

The World Health Organization states that about 438,000 and 620,000 people died from malaria in 2015 and 2017, respectively, whereas 300 to 500 million people are infected by malaria [1]. Malaria virus transmission is influenced by weather conditions that are suitable for a mosquito to live for extended periods, where environmental temperatures are high enough, particularly after rain. For that reason, 90% of malaria cases occur in Africa, and cases are also frequent in humid areas, such as Asia and Latin America [2,3,4]. If the disease is not treated at the early stages, this may even lead to death. The usual process for detecting malaria starts with collecting blood samples and counting the parasites and red blood cells (RBCs). Figure 1 shows images of RBCs both uninfected and infected by the malaria parasite. This process needs medical experts to collect and examine millions of blood samples, which is costly, time-consuming, and error-prone processes [5]. There are two traditional approaches for detecting malaria: one is very time-consuming because it needs to identify at least 5,000 RBCs, and another is an antigen-based fast diagnostic examination that is very costly. To overcome the limitations of the traditional approaches, in the last few years, researchers have focused on solving this problem using several machine learning and deep learning algorithms.

A number of studies have been carried out recently to identify malaria using image analysis by artificial intelligence (AI). Bibin et al. proposed a deep belief network (DBN) to detect malaria parasites (MPs) in RBC images [6]. They used 4100 images for training their model and achieved a specificity of 95.92%, a sensitivity of 97.60%, and an F-score of 89.66%. Pandit and Anand detected MPs from the RBC images using an artificial neural network [7] using 24 healthy RBC and 24 infected RBC images in order to train their model and obtained an accuracy of between 90% and 100%. Jain et al. used a CNN model to detect MPs from RBC images [8] without using GPU and preprocessing techniques while providing a low-cost detection algorithm, which achieved an accuracy of 97%. Rajaraman et al. pretrained CNN models for extracting the features from 27,558 RBC cell images to detect MPs and achieved an accuracy of 92.7% [5]. Alqudah et al. developed a lightweight CNN to accurately detect MPs using RBC images [9]. They trained their model using 19,290 images with 4134 test data and achieved an accuracy of 98.85%. Sriporn et al. used six transfer learning models (TL): Xception, Inception-V3, ResNet-50, NasNetMobile, VGG-16, and AlexNet to detect MPs [10]. Several combinations of activation function and optimizer were employed to improve the model’s effectiveness. A combined accuracy of 99.28% was achieved by their models trained with 7000 images. Fuhad et al. proposed an automated CNN model to detect MPs from RBC images [11] and performed three training techniques—general, distillation, and autoencoder training—to improve model accuracy after correctly labeling the incorrectly labeled images. Masud et al. proposed leveraging the CNN model to detect MPs using a mobile application [12] and a cyclical stochastic gradient descent optimizer and achieved an accuracy of 97.30%. Maqsood et al. developed a customized CNN model to detect MPs [13] with the assistance of bilateral filtering (BF) and image augmentation methods and achieved an accuracy of 96.82%. Umer et al. developed a stacked CNN model to predict MPs from thin RBC images and achieved an outstanding performance with an accuracy of 99.98%, precision of 100%, and recall of 99.9% [14]. Hung and Carpenter proposed a region-based CNN to detect the object from the RBC images [15]. The total accuracy using one-stage classification and two-stage classification was 59% and 98%, respectively. Pattanaik et al. suggested a methodology for detecting malaria from cell images using computer-aided diagnosis (CAD) [16]. They employed an artificial neural network with a functional link and sparse stacking to pretrain the system’s parameters and achieved an accuracy of 89.10% and a sensitivity of 93.90% to detect malaria from a private dataset of 2565 RCB pictures gathered from the University of Alabama at Birmingham. Olugboja et al. used a support vector machine (SVM) and CNN [17] to obtain accuracies of 95% and 91.66%, respectively. Gopakumar et al. created a custom CNN based on a stack of images [18]. A two-level segmentation technique was introduced after the cell counting problem was reinterpreted as a segmentation problem. An accuracy of 98.77%, a sensitivity of 99.14%, and a specificity of 99.62% were achieved from the CNN focus stack model.

Khan et al. used three machine learning (ML) models—logistic regression (LR), decision tree (DT), and random forest (RF)—to predict MPs from RBC images [19]. Firstly, they extracted the aggregated features from the cell images and achieved a high recall of 86% using RF. Fatima and Farid developed a computer-aided system (CAD) to detect MPs from RBC images [20] upon removing the noise and enhancing the quality of the images using the BF method. To detect the MPs, they used adaptive thresholding and morphological image processing and achieved an accuracy of 91%. Mohanty et al. used two models, autoencoder (AE) [21] and self-organizing maps (SOM) [22], to detect MPs and found that AE was better than SOM, which achieved an accuracy of 87.5% [23]. Dong et al. proposed three TL models, LeNet [24], AlexNet, and GoogLeNet [25], to detect MPs [26]. SVM was used to make a comparison with the TL models, which achieved an accuracy of 95%, which was more significant than the accuracy of 92% using the support vector machine (SVM). Anggraini et al. proposed a CAD to detect MPs from RBC images [27] with gray-scale preprocessing for stretching the contrast of the images and global thresholding to gain the different blood cell components from the images.

So far, many computerized systems have been proposed; most of them were based on traditional machine learning or conventional deep learning approaches, which provided satisfactory performances, but there is still scope for further improvement. After developing the vision transformer model [28], the attention-based transformer model has shown promising results in medical imaging, bioinformatics, computer vision tasks, etc. compared with the conventional convolution-based deep learning model. However, to date, no attention-based works have been carried out to detect malaria parasites. Again, the interpretability of a deep CNN model is a major issue. More recently, visualizing what a deep learning model has learned has attracted significant attention to the deep learning community. However, most previous works have failed to introduce the interpretability of the model for malaria parasite detection. To overcome these issues, in this work, an explainable transformer-based model is proposed to detect the malaria parasite from the cell image of blood smear images. Various hyperparameters, such as encoder depth, optimizer (Adam and stochastic gradient descent (SGD)), batch size, etc., were experimented with to achieve better performance. Two malaria parasite datasets (original and modified) were taken into consideration to conduct the experiments.

The key contributions of this paper are:

(1) A multiheaded attention transformer-based model was implemented for the detection of malaria parasites for the first time.
(2) The gradient-weighted class activation map (Grad-CAM) technique was applied to interpret and visualize the trained model.
(3) Original and modified datasets of malaria parasites were used for experimental analysis.
(4) The proposed model for malaria parasite detection was compared with SOTA models.

## 2. Proposed Methodology

**Figure 2:**
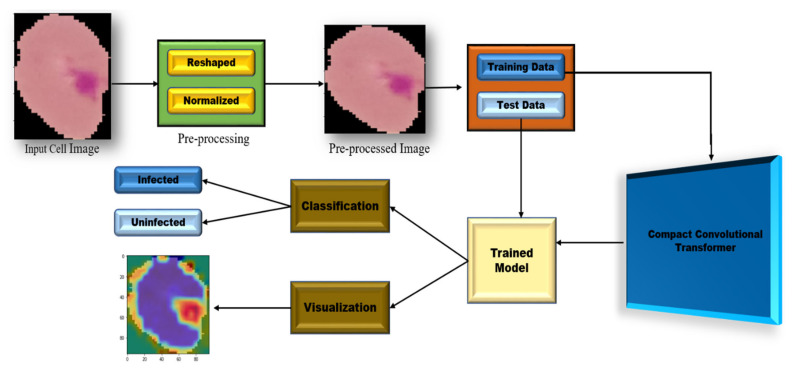
The overall design of the proposed methodology.

Figure 2 shows the overall design of the proposed methodology. Firstly, the raw images were preprocessed, followed by dataset splitting into training and testing sets to build the model. Finally, to visualize the trained model, Grad-CAM was used to show the heatmap image.

### 2.1. Dataset Description

The dataset for malaria detection contains segmented RBC images. It is archived at the National Library of Medicine and is also openly accessible at “https://lhncbc.nlm.nih.gov/LHC-publications/pubs/MalariaDatasets.html” (accessed on 10 May 2022). Rajaraman et al. [5] developed a segmentation process and implemented it for segmenting RBC images from thin blood smear images. The dataset has a total 27,588 RBC images, among which 13,779 are infected and 13,779 are uninfected images of the malaria parasite. A detailed distribution of the dataset is given in Table 1. This dataset was further studied by a medical expert in the research work conducted by Fuhad et al. [11]. They discovered some suspicious data in the dataset, including data that seemed to be infected but was labeled as uninfected, as well as data that appeared uninfected but was labeled as infected. The data that had been mislabeled was afterward manually annotated. These incorrectly labeled data were simply set aside during annotation, with 647 false infected and suspicious data and 750 false uninfected and suspicious data being eliminated. The updated dataset was uploaded to Google Drive [29], which is open to the public and also taken into account in this work. In both datasets, 20% of the images were used for testing purposes, and 80% of the images were used for training the proposed model.

### 2.2. Preprocessing (Resize)

The raw images of the dataset come in a variety of sizes. As the proposed model contains fully connected layers in the classifier layer, the model needs a fixed-sized input image [30]. Therefore, the raw images were resized into 96 × 96 × 3. 

### 2.3. Model Architecture

**Figure 3:**
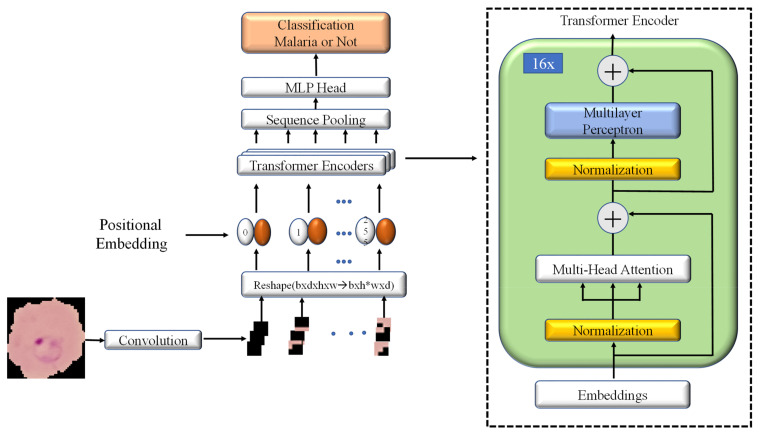
Compact convolutional transformer (CCT) model architecture.

Various attention-based models have been developed recently. To date, the vision transformer has most attracted researchers for computer vision tasks [28]. The compact convolutional transformer (CCT) is a slightly modified model from the vision transformer introduced in 2021 [31]. CCT with Grad-CAM visualization was implemented in this study to detect the malaria parasite. Figure 3 shows the model architecture of CCT.

#### 2.3.1. Convolutional Block

The traditional convolutional layer and the ReLU(.) activation function was used. A 3 × 3 kernel size with stride 3 was used to make it a nonoverlapping slide. After that, a maxpool layer was used. Instead of a full input image in the transformer model, the input image is divided into patches/grid images, which are given to the transformer’s encoder. In the proposed transformer-based model, the convolution filter was used for patching. Instead of patching images directly, these convolutional blocks took the input images to a latent representation that provides more flexibility than the vision transformer. Filters for the convolutional layer were employed to align with the vision transformer embedding dimension. Given an input image $X\in {\mathbb{R}}^{H\times W\times C}$

(1) $$X^{\prime }=MaxPool\left(ReLU\left(Conv2d\left(X\right)\right)\right)\in {\mathbb{R}}^{H^{\prime }\times W^{\prime }\times E}$$

where *E* is the number of filters = 768.

After the convolutional layer, the output image was reshaped from ${\mathbb{R}}^{H^{\prime }\times W^{\prime }\times E}$ to ${\mathbb{R}}^{N\times E}$ for converting it to the convolutional patches, where the number of sequences or patches $N\equiv \left(H^{\prime }W^{\prime }\right)$. This convolutional block maintains the locally spatial information. To keep tracking the position or sequence number of each patch, a learnable positional embedding was added.

#### 2.3.2. Multiheaded Attention Mechanism

**Figure 4:**
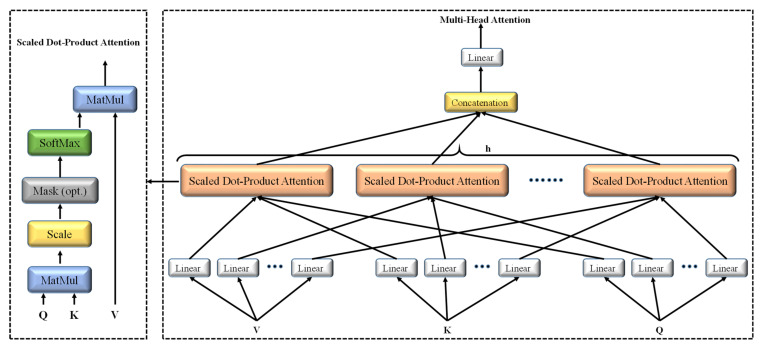
Scaled dot-product-based multihead attention mechanism.

The main part of the compact convolutional transformer is the multiheaded self-attention (MSA). The whole part of an image is not necessary for extracting valuable information; the attention mechanism focuses on the valuable part. Various attention mechanisms have been developed so far. However, the multiheaded self-attention was first introduced in the vision transformer. Figure 4 shows the scaled dot-product-based multihead attention mechanism [32].

The input is projected to queries, keys, and values using different learnable weights with linear layers in self-attention.

(2) $$Q=(X^{\prime }\in {\mathbb{R}}^{N\times E})\times \left(W_{Q}\in {\mathbb{R}}^{E\times d}\right)$$

(3) $$K=(X^{\prime }\in {\mathbb{R}}^{N\times E})\times \left(W_{K}\in {\mathbb{R}}^{E\times d}\right)$$

(4) $$V=(X^{\prime }\in {\mathbb{R}}^{N\times E})\times \left(W_{V}\in {\mathbb{R}}^{E\times d}\right)$$

Now queries,$\;Q\in {\mathbb{R}}^{N\times d}$; keys, $K\in {\mathbb{R}}^{N\times d}$; and values, $V\in {\mathbb{R}}^{N\times D}$. In the case of a scaled dot-product form of attention, the dot product is computed between the queries and keys, which is scaled by $\surd d_{k}$. After that, nonlinear softmax function is imposed to obtain the attention weights.

(5) $$Z^{\prime }=\left(Q\in {\mathbb{R}}^{N\times d}\right)\times \left(K^{T}\in {\mathbb{R}}^{d\times N}\right)$$

(6) $$Z=softmax\left((Z^{\prime }\in {\mathbb{R}}^{N\times N}\right)/\surd d_{K})$$

This $Z\in {\mathbb{R}}^{N\times N}$ is the attention weight among the patches. This attention weight is then multiplied with values to obtain the self-attention weighted output $H\in {\mathbb{R}}^{N\times d}$

(7) $$H=\left(Z\in {\mathbb{R}}^{N\times N}\right)x\;\left(V\in {\mathbb{R}}^{N\times d}\right)$$

Therefore, the scaled dot-product attention function can be written in shorted form as below:

(8) $$Attention\left(Q,\;K,\;V\right)=softmax\left(\frac{QK^{T}}{\surd d_{K}}\right)V$$

Rather than performing a single attention function with d-dimensional queries, keys, and values, it is advantageous to linearly project queries, keys, and values to *d_h_*, *d_k_*, and *d_v_* dimensions, *h* times using different learnable weights with linear layers. After that, the scaled dot-product attention function is applied in parallel in all the h heads, resulting in h number of d_*v*_-dimensional values. These attention-weighted values are then concatenated and further projected with linear layers. The multiheaded attention mechanism helps the model attend to different parts from different representation subspaces. In this work, *d_h_* = *d_k_* = *d_v_* = *d* was applied, and the number of heads used was *h* = 8.

(9) $$head_{i}=Attention\left(Q,\;K,\;V\right)\in {\mathbb{R}}^{N\times d}$$

(10) $$MultiHead\left(Q,\;K,\;V\right)=Concatenate\left[head_{1},\;head_{2},\ldots head_{h}\right]W^{0}$$

where $W^{0}\in {\mathbb{R}}^{hd\times d}$.

#### 2.3.3. Transformer Encoder

The image patches come from the convolutional block and are passed through the transformer encoder. Firstly, layer normalization is applied to the image patches that normalize the activations along the feature direction instead of the mini-batch direction in batch normalization. Then multiheaded self-attention is applied to these normalized patches. The results are added with the residual connected original patches, as shown in Figure 4. Further layer normalization and a feed-forward block are imposed along with another residual connection. The feed-forward block has two linear layers along with a dropout layer and a GELU nonlinearity. The first linear layer expands the dimension four times, and the second linear layer reduces the dimension back (feed-forward block).

(11) $$O\prime =Linear\left(A\in {\mathbb{R}}^{N\times d}\right)\in {\mathbb{R}}^{N\times 4d}$$

(12) $$O^{\prime \prime }=Dropout\left(GELU\left(O^{\prime }\right)\right)\in {\mathbb{R}}^{N\times 4d}$$

(13) $$O=Linear\left(O^{\prime \prime }\in {\mathbb{R}}^{N\times 4d}\right)\in {\mathbb{R}}^{N\times d}$$

The outcomes from these two paths are added again. For this work, 16 transformer encoders were implied sequentially.

#### 2.3.4. Sequence Pooling

In the vision transformer, a class token is used to classify the final output. However, in the compact convolutional transformer, instead of using a class token, sequence pooling is used. Sequence pooling pools over the entire sequence of data. Given the output of the last transformer encoder block $X_{L}\in {\mathbb{R}}^{b\times N\times d}$, *b* is the mini-batch size, *N* is the number of sequences, $X_{L}$ is sent through a linear layer, and then the output $X_{L}^{\prime }$ from this linear layer is multiplied with $X_{L}$.

(14) $$X_{L}^{\prime }=\in softmax\left(Linear\left(X_{L}\in {\mathbb{R}}^{b\times N\times d}\right)\right)\in {\mathbb{R}}^{b\times N\times 1}$$

(15) $$F=\left(X_{L}^{\prime }\in {\mathbb{R}}^{b\times N\times 1}\right).\left(X_{L}\in {\mathbb{R}}^{b\times N\times d}\right)\;=\left(X_{L}^{\prime T}\in {\mathbb{R}}^{b\times 1\times N}\right)\left(X_{L}\in {\mathbb{R}}^{b\times N\times d}\right)\;=Reshape\left(X_{L}^{\prime \prime }\in {\mathbb{R}}^{b\times 1\times d}\right)\in {\mathbb{R}}^{b\times d}$$

The output *F* is then sent to the final linear classifier to classify the input data.

## 3. Grad-CAM Visualization

The gradient-weighted class activation map (Grad-CAM) is a technique to interpret what the model has actually learned [33]. This technique generates a class-specific heatmap using a trained deep learning model for a particular input image. This Grad-CAM approach highlights the input image regions where the model pays much attention to producing discriminative patterns from the last layer before the final classifier, as the last layer contains the most highly semantic features. Grad-CAM uses the feature maps from the last convolutional layer, providing the best discriminative semantics. Let *y^c^* be the class score for class c from the classifier before the SoftMax layer. Grad-CAM has three basic steps:

Step-1: Compute the gradients of class score y^C^ with respect to the feature maps A^k^ of the last convolutional layer before the classifier, i.e.,

$$\frac{\partial y^{c}}{\partial y^{k}}\in {\mathbb{R}}^{F\times U\times V}$$

where the feature map is

$$A^{k}\in {\mathbb{R}}^{F\times U\times V}$$

Step-2: To obtain the attention weights *α^c^*, global average pool the gradients over the width (indexed by i) and height (indexed by j).

(16) $$\alpha_{k}^{c}=\frac{1}{Z}\underset{i}{\sum }\underset{j}{\sum }\frac{\partial y^{c}}{\partial A_{ij}^{k}}\;=\in {\mathbb{R}}^{F\times 1\times 1}\;\;=\in {\mathbb{R}}^{F}\left[simplify\right]$$

Step-3: Calculate the final Grad-CAM heatmap by the weighted (*α^c^*) sum of feature maps (*A^k^*) and then apply the ReLU (.) function to retain only the positive values and turn all the negative values into zero.

(17) $$L_{heatmap}^{c}=ReLU\left(\underset{k}{\sum }\alpha_{k}^{c}A^{k}\right)\;=\in {\mathbb{R}}^{U\times V}$$

**Figure 5:**
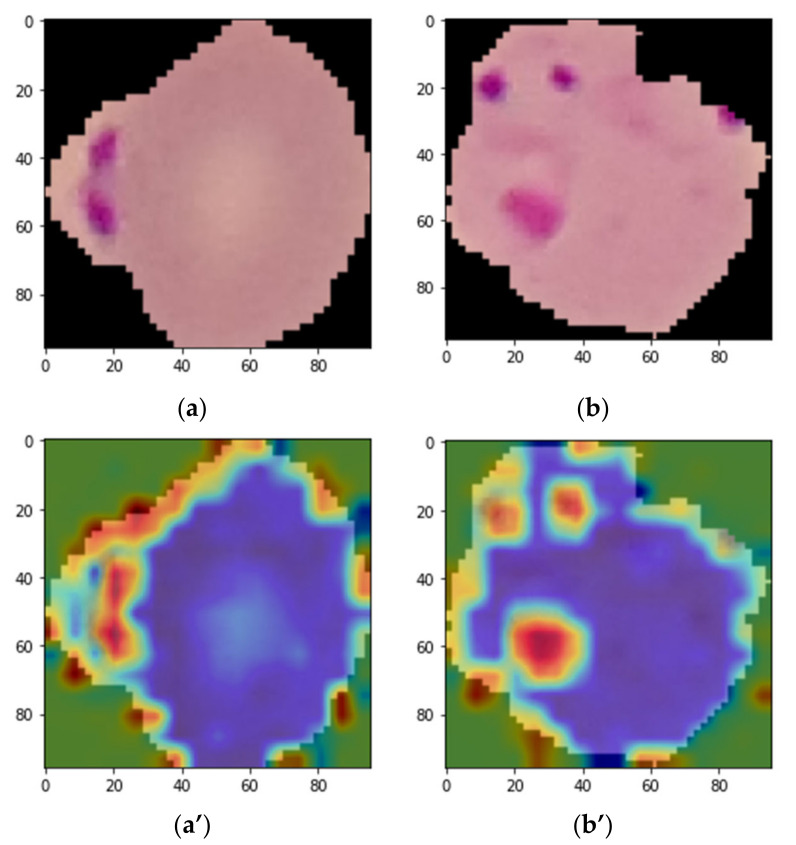
Grad-CAM localization map of the input images (**a**,**b**) and their corresponding overlaid heat map (**a**’,**b**’).

Firstly, the proposed model was trained with the training samples from the dataset. After the training phase was completed, the trained model was used for evaluation with the testing parts of the dataset. In addition, to explain what the trained model had actually learned, the Grad-CAM technique explained above was applied. Various test images were selected randomly to generate the corresponding heatmap from the trained model using the Grad-CAM approach. In this case, the multilayer perceptron layer of the last transformer encoder before the final classifier was chosen as the target layer. Features and gradients were extracted from that layer, and a heatmap was generated using the above Grad-CAM formula. Subsequently, the heatmap was resized with nearest-neighbor interpolation as the same size as the input image, and the heatmap was overlaid with the input image. Figure 5 shows the original input images and their corresponding heatmap images. For the heatmap image conversion, a jet color map was used. It can be seen from the overlaid heatmap images that the lesion areas are much more reddish than the other regions of the image. These reddish areas are the main lesions responsible for the malaria parasite [34].

There is no existing segmentation dataset of RBC cell images with the parasite mask on the RBC cell image for quantitative analysis. Annotation made in the dataset used in this work was that normal RBC images come with a clean version without any lesions, but the parasite images come with lesions [34]. Based on the presence of these lesions, the RBC cell images were classified either as normal or parasite. To show explainability of the trained model, the CAM technique was applied to generate heatmap images that showed the actual parts (lesions) the model paid attention to during feature extraction and classification. This technique can bring new insights toward detecting MPs accurately.

## 4. Result Analysis

### 4.1. Performance Evaluation Procedure

Pytorch python framework was used to conduct the whole experiment. The model was run on a highly computing GPU-supported Desktop PC with 11th Generation Intel (R) Core (TM) i9-11900 CPU @2.50GHz, 32 GB RAM, NVIDIA GeForce, and RTX 3090 24 GB GPU running on a 64-bit Windows 10 Pro operating system.

The cell images were preprocessed, then the proposed transformer-based model was trained using original and modified datasets. The performance of the model was tested using 20% of the dataset in both cases. In both cases, the proposed model was trained for 50 epochs, and the learning rate was fixed to 0.001. Various hyperparameters such as optimizers, batch size, and transformer’s encoder depth were experimented with for performance analysis.

For measuring the performance of the deep learning model, various evaluation metrics were used. The proposed work was evaluated with confusion matrix (CM), accuracy, precision, recall, f1-score, and area under the curve (AUC) [35,36]:

(18) $$Accuracy=\frac{T_{P}+T_{N}}{T_{P}+T_{N}+F_{P}+F_{N}}$$

(19) $$Recall=\frac{T_{P}}{T_{P}+F_{N}}$$

(20) $$Precision=\frac{T_{P}}{T_{P}+F_{P}}$$

(21) $$AUC=\frac{1}{2}\left(\frac{T_{P}}{T_{P}+F_{N}}+\frac{T_{N}}{T_{N}+F_{P}}\right)$$

where *T_P_* = true positive means that a malaria-infected person is correctly detected as a malaria-infected person, *T_N_* = true negative means that a noninfected person is correctly detected as a noninfected person, *F_P_* = false positive means that a noninfected person is wrongly detected as an infected person, and *F_N_* = false negative means that an infected person is wrongly detected as a noninfected person.

The original and modified datasets were considered for balanced binary classification. To examine the performance of the proposed model, various hyperparameters were considered. Among various optimization methods developed so far for deep learning, “Adam” [37] and “SGD” [38] are the two most used and popular optimization ones. Therefore, to demonstrate their effectiveness in malaria parasite detection, the proposed model was trained using both optimizers. Batch size is also a key factor for the model’s learning and obtaining more generalized results. A larger batch size makes a model speed up the training process, whereas a much larger batch size very often provides poor generalization. In this study, the proposed model was also tuned with various batch sizes (8, 16, 32, and 64). Furthermore, different encoder depths (8, 12, and 16) were also experimented with.

### 4.2. Results Obtained with Original Dataset

#### 4.2.1. Adam Optimizer for Original Dataset

**Figure 6:**
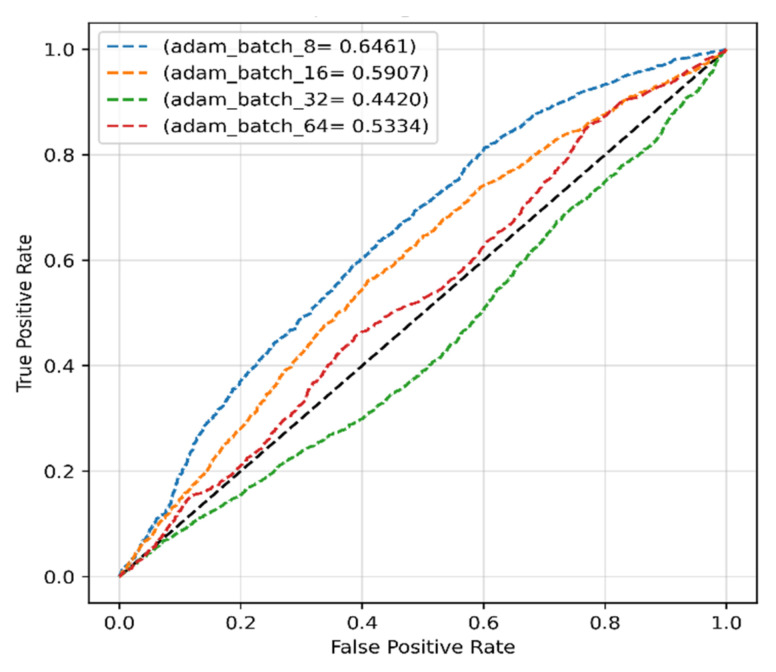
The ROCs of the proposed model obtained with original dataset and ADAM optimizer.

The different performance criteria of the proposed model with the ADAM optimizer, for instance, precision, recall, F1-score, and accuracy, were calculated and are presented in Table 2. The ROCs of the model with the ADAM optimizer for different batch sizes are presented in Figure 6, which shows that the highest AUC of 64.61% was achieved using a batch size of 8. This could be due to the fact that the model trained with larger batch sizes with the Adam optimizer did not show a continuous improvement. Even though the ADAM optimizer produced very high precision, the other results for recall, F1-score, and accuracy were disappointing.

#### 4.2.2. SGD Optimizer for Original Dataset

**Figure 7:**
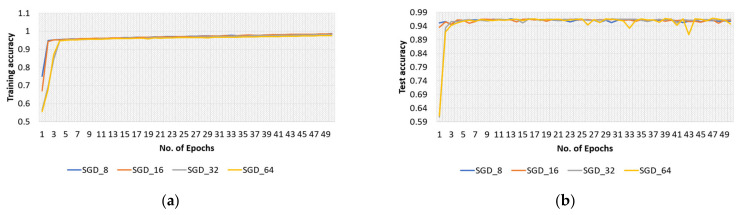
Accuracy curves of (**a**) training and (**b**) test phases of the proposed model obtained with original dataset and SGD optimizer.

**Figure 8:**
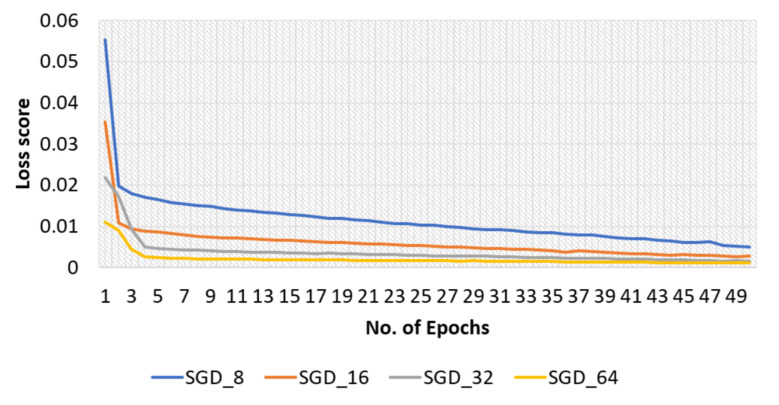
Loss curve of training phase of the proposed model obtained with original dataset and SGD optimizer.

The effectiveness of the model with the SGD optimizer, along with various batch sizes, is briefly discussed in this section. The model’s training and test accuracies for the original dataset are shown in Figure 7, and the training loss curve in Figure 8.

**Figure 9:**
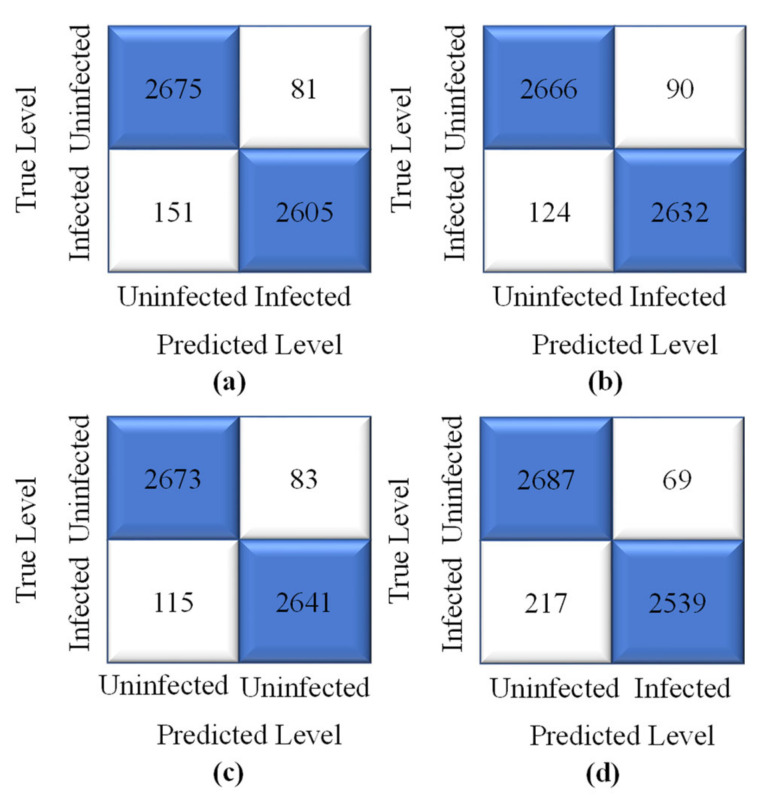
Confusion matrix of the proposed model obtained with original dataset, SGD optimizer, and batch sizes of (**a**) 8 (**b**) 16 (**c**) 32, and (**d**) 64.

The proposed model’s highest training accuracy was 98.61%, which was achieved with a batch size of 8, whereas the highest testing accuracy was 96.86% with a batch size of 64. A batch size of 64 resulted in the shortest training loss of 0.1%. To calculate how well the proposed model with the SGD optimizer detects malaria-infected patients, the same number of cell images as in Adam was used for testing. A number of predicted patients are shown by the CM in Figure 9. The highest accuracy of 96.41% was achieved with the SGD optimizer and a batch size of 32, and the highest recall of 95.88% was achieved for the same batch size (Table 3).

**Figure 10:**
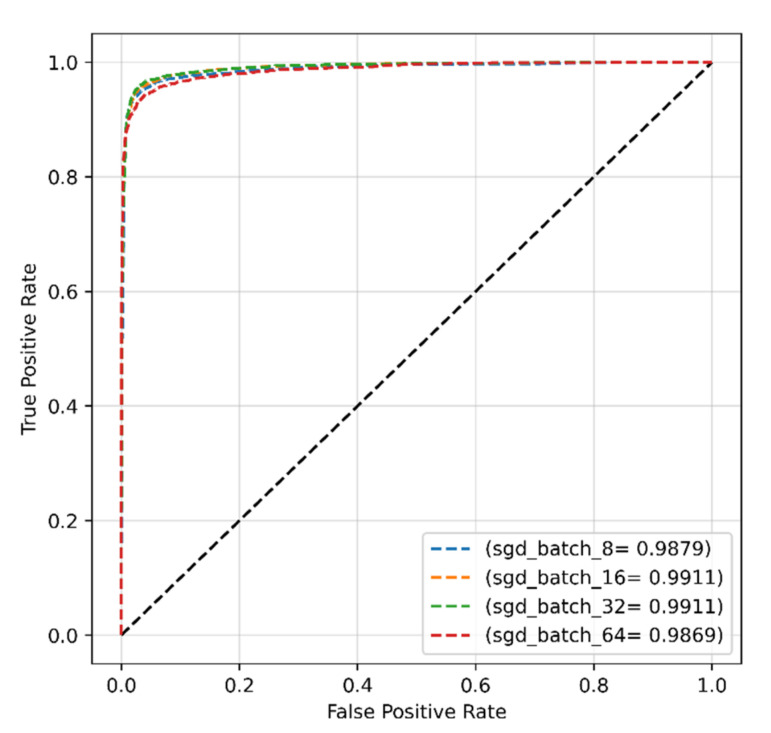
The ROC of the proposed model obtained with original dataset and SGD optimizer.

Figure 10 shows the ROCs of the proposed model with the SGD optimizer for various batch sizes, and the results indicated the greatest AUC of 99.11% was reached with batch sizes of 16 and 32.

#### 4.2.3. Encoder Depth for Original Dataset

**Figure 11:**
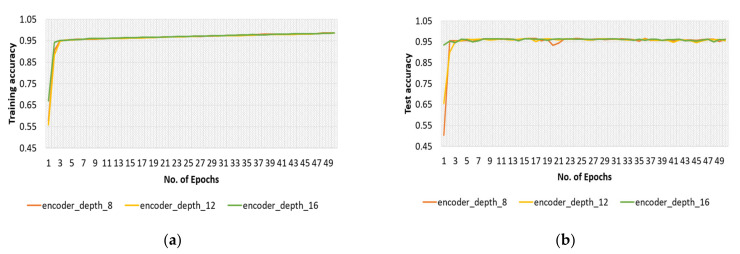
(**a**) Training curves and (**b**) test curves of the proposed model obtained with original dataset and encoder depths of 8, 12, and 16.

To show the impact of different depths of the encoder, the SGD optimizer was used, and the batch size was fixed to 16. The highest test accuracy of 96.66% was obtained with an encoder depth of 8 (Figure 11).

**Figure 12:**
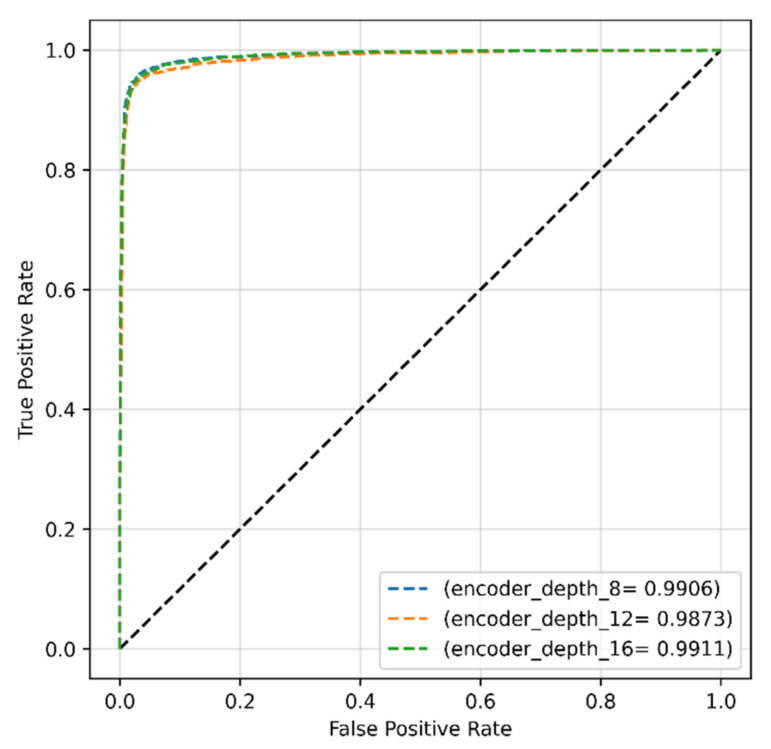
ROC curves of the proposed model obtained with original dataset and encoder depths of 8, 12, and 16.

ROC scores of ~99% were achieved from the model with all encoder depths of 8, 12, and 16 (Figure 12).

### 4.3. Results Obtained with Modified Dataset

#### 4.3.1. Adam Optimizer for Modified Dataset

**Figure 13:**
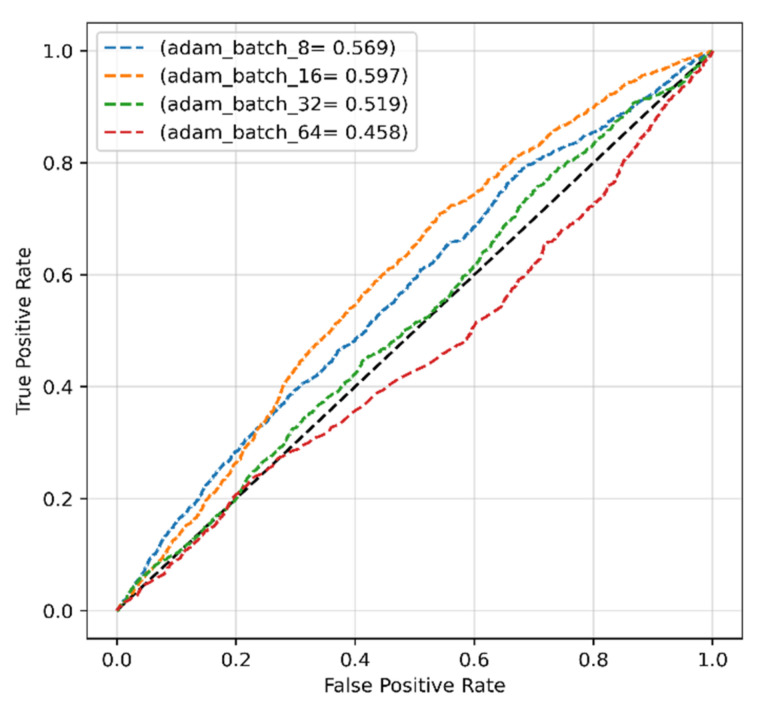
The ROC of the proposed model obtained with modified dataset and ADAM optimizer.

The proposed transformer-based model’s classification performance with the modified dataset and Adam optimizer was evaluated and is presented in Table 4. Again, other than precision, the other performance results were poor. The highest ROC of 59.7% was achieved with a batch size of 16 (Figure 13), indicating no promising results.

#### 4.3.2. SGD Optimizer for Modified Dataset

**Figure 14:**
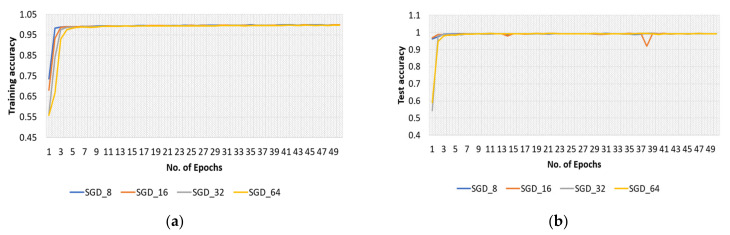
Accuracy curves of (**a**) training and (**b**) test phases of the proposed model obtained with modified dataset and SGD optimizer.

**Figure 15:**
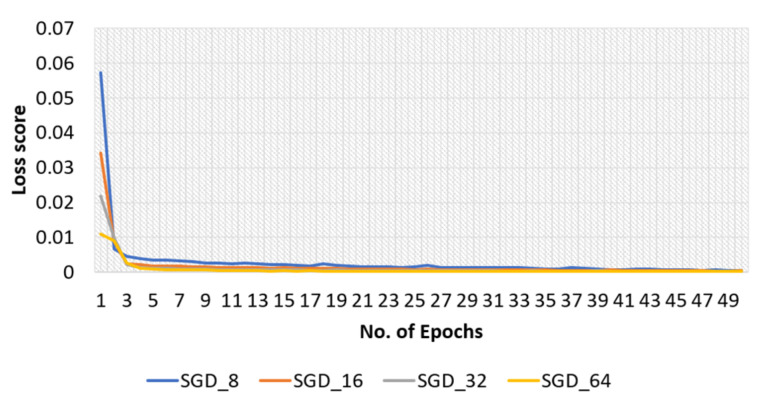
Loss curve of training phase with modified dataset, SGD optimizer, and batch sizes of 8, 16, 32, and 64.

Furthermore, the SGD optimizer was used with various batch sizes for the modified dataset, and accuracy and loss curves are shown in Figure 14 and Figure 15, respectively. 

**Figure 16:**
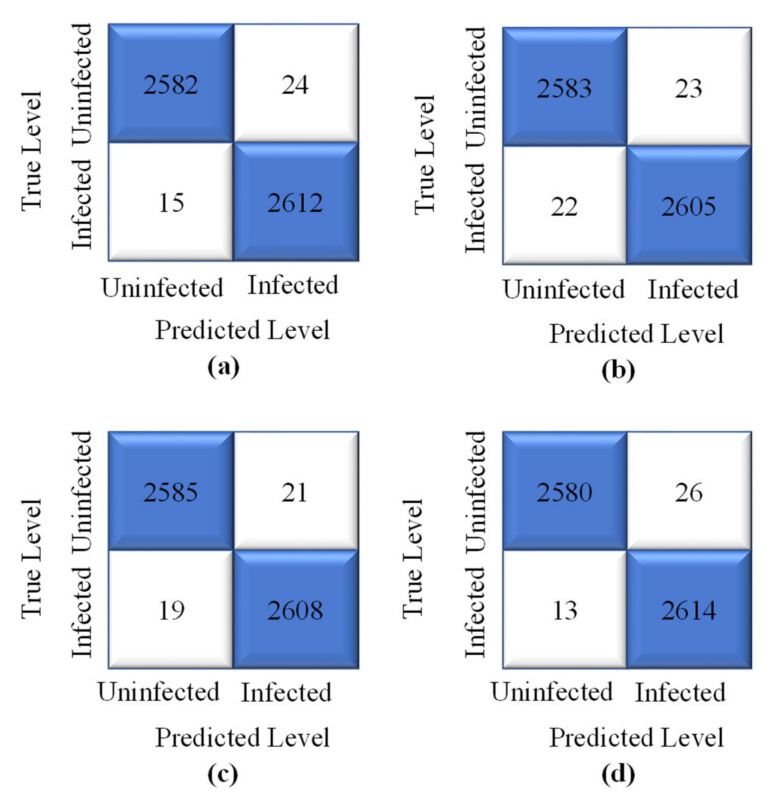
Confusion matrix of the proposed model obtained with modified dataset, SGD optimizer, and batch sizes of (**a**) 8, (**b**) 16, (**c**) 32, and (**d**) 64.

**Figure 17:**
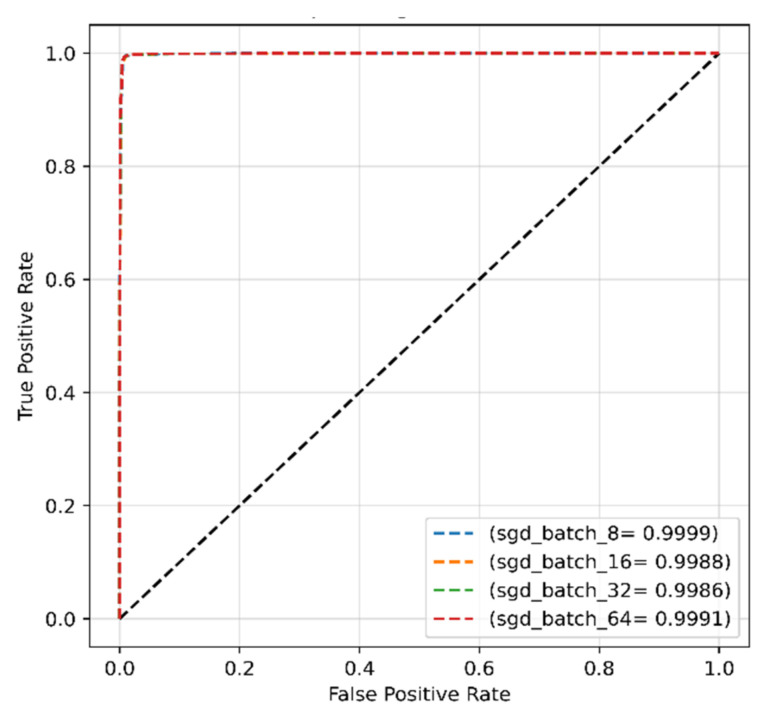
The ROC of the proposed model obtained with SGD optimizer and modified dataset.

The CMs for the model with the SGD optimizer are shown in Figure 16. Although the highest accuracy of 99.25% and recall of 99.50% were achieved for a batch size of 64 with the SGD optimizer, the results showed insignificant differences between the batch sizes (Table 5). The ROCs of each batch size are demonstrated in Figure 17; the results did not show much difference, with AUC values close to 1.0 in all cases.

#### 4.3.3. Encoder Depth for Modified Dataset

**Figure 18:**
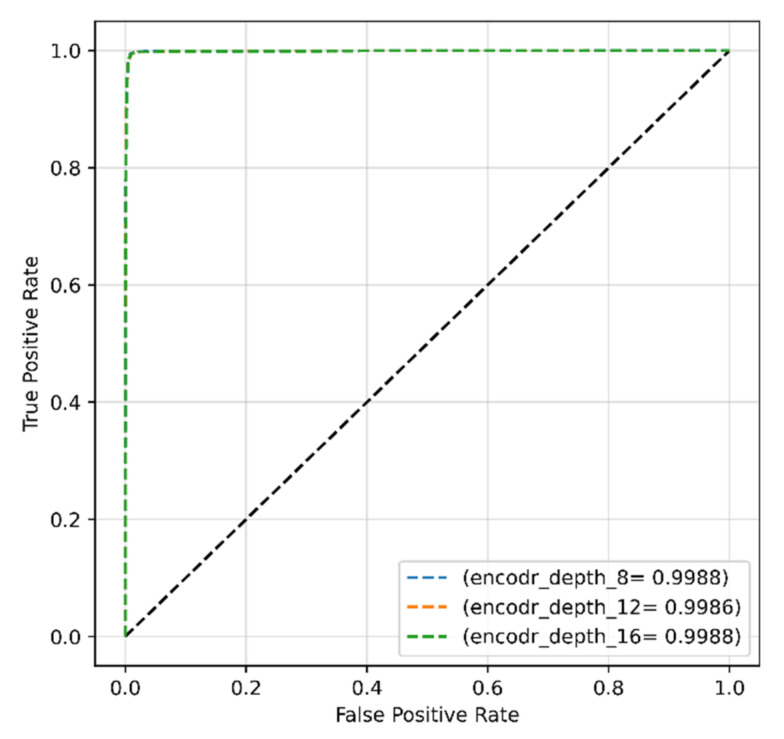
ROC curves of the proposed model obtained with modified dataset and encoder’s depth of 8, 12, and 16.

Encoder depth was also finetuned for the modified dataset. The larger model with higher encoder depth showed higher fluctuation, and the highest test accuracy of 99.29% was obtained from the proposed model with encoder depths of 8 and 16. ROC curves in Figure 18 ensured that all models achieved the same high AUC score of approximately 99.9%.

### 4.4. Performance Comparison between Two Datasets

**Figure 19:**
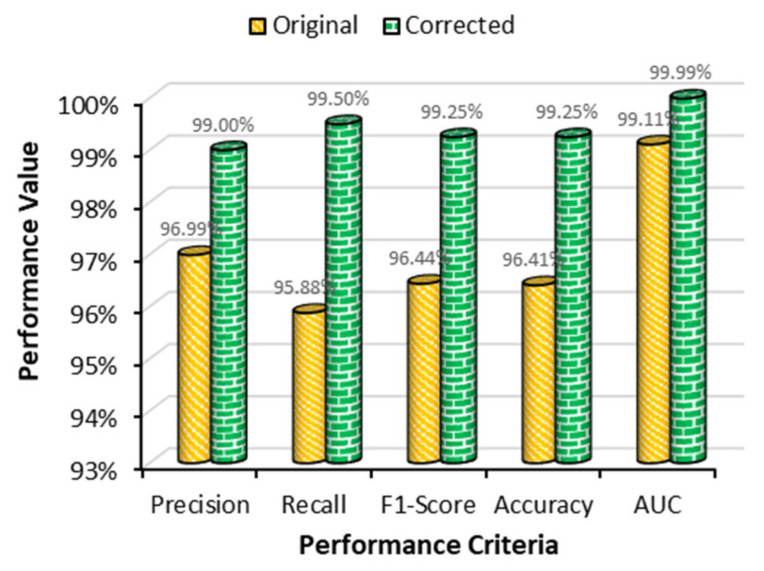
Performance comparison of original and modified datasets for the transformer-based model with SGD optimizer.

With smaller batch sizes, the learning process became easier and provided the best evaluation results, but on the other hand, with greater batch sizes, the transformer-based model converged faster and provided much more generalization. After correcting the mislabeled data in the original datasets, the problem became a much easier balanced binary classification. From the above experimental results, it was observed that the Adam optimizer showed poor results in all cases, as it was not guaranteed to converge to the global optimum point. On the other hand, the results obtained using the SGD optimizer were significantly better than that obtained by the Adam optimizer for all performance criteria employed in this work. From Figure 19, it was observed that the classification performance of the model was optimistic with the modified dataset rather than the original dataset. Therefore, the proposed model with the SGD optimizer and modified dataset could be the best combination for accurate MP detection.

However, a similar comparison made between the two datasets using the Adam optimizer showed no improvement with the modified dataset. Furthermore, this indicated that for both datasets, the SGD optimizer could produce optimistic results.

### 4.5. Performance Comparison with Previous Works

In this section, the performance of the proposed model is compared with several SOTA methods for both datasets. The details of the SOTA methods have been described in the Introduction section.

For the original dataset, the first five rows show the results of the SOTA models in Table 6. It was observed that the highest accuracy score of 91.80% was achieved from the previous work by Fatima and Farid [20]. On the other hand, the proposed transformer-based model achieved a promising accuracy of 96.41% when the SGD optimizer was used with a batch size of 32 and a learning rate of 0.001, almost 5% higher than the other best work reported to date. This suggests that the proposed model could produce even better results than that reported using the same dataset.

For the modified dataset, the highest accuracy of 99.23% was achieved by Fuhad et al. [11]. However, the AUC of the SOTA models did not achieve a satisfactory level, whereas the proposed model showed an optimistic AUC score of 99.99% when the SGD optimizer with a batch size of 64 and a learning rate of 0.001 was employed. The highest AUC of the proposed model proved the highest differentiation capability between malaria-infected and uninfected patients. The table suggested that the proposed transformer-based model achieved a satisfactory classification performance compared with the SOTA models mentioned.

RBC images were collected from an open-source repository. However, the real working procedure starts with segmenting, first, the RBCs in blood smear images that contain various other cells. Afterward, from the affected regions of the RBCs, the malaria parasite is classified. In this work, the segmentation task was ignored, and readymade RBC cell images were used only to classify the malaria parasite. Conventional CNN models show image-specific inductive bias [40], and they are based on a local receptive field. To capture global information, CNN models need larger kernels or very deep network models. However, the transformer models are free from these shortcomings, and, therefore, the transformer-based model proposed for the malaria parasite showed excellent performance. Moreover, the Grad-CAM visualization demonstrated its explanation visibility. It was also noticed that similar to this study, the application of the SGD optimizer by other studies also produced the highest performance.

## 5. Conclusions

A multiheaded attention-based transformer model was proposed for malaria parasite detection. In addition, to interpret the trained model, Grad-CAM visualization was used to verify the learning. The proposed work with the transformer model achieved accuracy, precision, recall, and an AUC score of 99.25%, 99.00%, 99.50%, and 99.99%, respectively. Various SOTA works for malaria parasite detection were compared with the proposed model. The model outperformed all the previous works for detecting malaria parasites. Our future work will be focused on segmenting RBCs from blood smear images and classifying malaria parasites from the segmented RBC images.

## Figures and Tables

**Table 1:** Data distribution in the dataset used in this work.

Dataset	Number of Healthy Images	Number of Infected Images	Total	Total Training Samples (80%)	Total Testing Samples (20%)
Original dataset [5]	13,779	13,779	27,558	22,046	5512
Modified dataset [11]	13,029	13,132	26,161	20,928	5233

**Table 2:** Model’s performance for various batch sizes with ADAM optimizer and original dataset.

Batch Size	Precision (%)	Recall (%)	F1-Score (%)	Accuracy (%)
8	52.10	**62.03**	56.64	**60.11**
16	63.17	56.05	59.40	56.82
32	100	50	**66.67**	50
64	**99.96**	49.99	66.65	49.98

Note: Bold numbers indicate highest value within a column.

**Table 3:** Model’s performance for various batch sizes with SGD optimizer and original dataset.

Batch Size	Precision (%)	Recall (%)	F1-Score	Accuracy (%)
8	97.06	94.66	95.84	95.79
16	96.73	95.56	96.14	96.12
32	96.99	**95.88**	**96.44**	**96.41**
64	**97.50**	92.53	94.95	94.81

Note: Bold numbers indicate highest value within a column

**Table 4:** Model’s performance for various batch sizes with ADAM optimizer and modified dataset.

Batch Size	Precision (%)	Recall (%)	F1-Score (%)	Accuracy (%)
8	58.37	53.58	55.87	54.08
16	48.12	**59.69**	53.28	**57.98**
32	100	49.80	66.49	49.80
64	**100**	49.80	**66.49**	49.80

Note: Bold numbers indicate highest value within a column.

**Table 5:** Model’s performance for various batch sizes with SGD optimizer and modified dataset.

Batch Size	Precision (%)	Recall (%)	F1-Score (%)	Accuracy (%)
8	99.08	99.42	**99.25**	**99.25**
16	99.12	99.16	99.14	99.14
32	**99.19**	99.27	99.23	99.24
64	99.00	**99.50**	**99.25**	**99.25**

Note: Bold numbers indicate highest value within a column.

**Table 6:** Performance comparison with previous works.

Reference No	Model Used	Optimizer	Learning Rate	Batch Size	Precision (%)	Recall (%)	AUC (%)	Accuracy (%)
[39]	Custom CNN	Adam	-	-	-	-	-	95
[20]	Image processing	-	-	-	94.66	-	-	91.80
[19]	Random forest	-	-	-	82.00	86.00	-	-
[5]	CNN	SGD	0.0005	-	94.70	**95.90**	**99.90**	-
[16]	Neural network	-	-	-	93.90	-	-	83.10
Proposed work (original dataset)	Transformer	SGD	0.001	32	**96.99**	95.88	99.11	**96.41**
[9]	CNN	Adam	0.001	128	98.79	-	-	98.85
[11]	Custom CNN	SGD	0.01	32	98.92	**99.52**	-	99.23
Proposed work (modified dataset)	Transformer	SGD	0.001	32	**99.00**	99.50	**99.99**	**99.25**

Note: Bold numbers indicate highest value within a column.

## Data Availability

The data presented in this study are available in the article.
